# Soft power and global health: the sustainable development goals (SDGs) era health agendas of the G7, G20 and BRICS

**DOI:** 10.1186/s12889-019-7114-5

**Published:** 2019-06-24

**Authors:** Bronwyn McBride, Sarah Hawkes, Kent Buse

**Affiliations:** 1 Interdisciplinary Studies Graduate Program, University of British Columbia, 270, 2357 Main Mall, H. R. MacMillan Building, Vancouver, BC V6T 1Z4 Canada; 2 Institute for Global Health, University College London, 30 Guilford Street, London, WC1N 1EH UK; 3 Strategic Policy Directions, UNAIDS, Avenue Appia 20, 1211 Geneva, Switzerland

**Keywords:** Global health diplomacy, Soft power, Global health agenda-setting, Sustainable development goals, BRICS, G7, G20

## Abstract

**Background:**

In 2017, the G20 health ministers convened for the first time to discuss global health and issued a communiqué outlining their health priorities, as the BRICS and G7 have done for years. As these political clubs hold considerable political and economic influence, their respective global health agendas may influence both global health priorities and the priorities of other countries and actors.

**Methods:**

Given the rising salience of global health in global summitry, we analyzed the health ministerial communiqués issued by the BRICS, G7 and G20 after the SDGs were adopted in 2015. We compared the stated health priorities of the BRICS, G7 and G20 against one another and against the targets of SDG 3 on health, using a traffic light system to assess the quality of their commitments.

**Results:**

With regard to the SDG 3 targets, the BRICS, G7 and G20 priorities overlapped in their focus on emergency preparedness and universal health coverage, but diverged in areas of environmental pollution, mental health, and maternal and child health. Health issues with considerable associated burdens of disease, including substance use, road traffic injuries and sexual health, were missing from the agendas of all three political clubs. In terms of SDG 3 principles and ways of working, the BRICS, G7 and G20 varied in their emphasis on human rights, equity and engagement with non-state actors, but all expressed their explicit commitment to Agenda 2030.

**Conclusions:**

The leadership of BRICS, G7 and G20 on global health is welcome. However, their relatively narrow focus on the potential impact of ill-health primarily in relation to the economy and trade may not be sufficiently comprehensive to achieve the Agenda 2030 vision of promoting health equity and leaving no-one behind. Recommendations for the BRICS, G7 and G20 based on this analysis include: 1) expanding focus to the neglected SDG 3 health targets; 2) placing greater emphasis on upstream determinants of health; 3) greater commitment to equity and leaving no-one behind; 4) adopting explicit commitments to rights-based approaches; and 5) making commitments that are of higher quality and which include time-bound quantitative targets and clear accountability mechanisms.

## Background

The G20 consists of the 20 most powerful economies – covering 85% of global gross domestic product and around two thirds of the world’s population, including over half of the world’s economically poor [1]. Billed as “a leading forum of the world’s major economies that seeks to develop global policies to address today’s most pressing challenges”, meetings of the G20 carry significant implications for the direction of economic and development policy globally. Following the first ever discussion of global health by the G20 health ministers in 2017, Richard Horton of The Lancet concluded that its ensuing Declaration’s promises were “platitudes” that lacked “concrete and specific actions” [2]. Global health scholar Ilona Kickbusch defended the effort, arguing that a door had been opened to include global health on the collective agenda of the major world economies [3], and the WHO emphasized strengthening health diplomacy through work with the BRICS, G7 and G20 in the General Programme of Work 2019–2023 presented at the 71st World Health Assembly in May 2018 [4] .

The G20 are not alone in their interest in global health. Members of the G20 also belong to the G7 (Canada, France, Germany, Italy, Japan, the United Kingdom and the United States) and BRICS (Brazil, Russia, India, China, and South Africa). Since the adoption of the Sustainable Development Goals (SDGs) in September 2015, both the BRICS and G7 have also made global health commitments at their heads of states summits and health ministerial meetings [5, 6]. As the members of these three political “clubs” [7–9] hold the lion’s share of global political and economic influence [10–15] as well as development assistance for health [8, 11, 16–19], the health issues prioritized by the BRICS, G7 and G20 through global health diplomacy matter. Through a confluence of soft power (defined as persuasion through the force of ideas, knowledge and cultural values) [9, 11, 20] and hard power (coercion through political, economic and military might) [11] combined in mutually reinforcing ways, their expressions can influence the health agendas (e.g., universal health coverage [UHC]) [11, 21] and health priorities (e.g., access to essential medicines, tobacco control) [11, 18, 19, 22] of both ‘recipient’ countries and other global health actors (i.e., the World Bank, GAVI Vaccine Alliance) [8, 9, 15, 23, 24](Table 1).

**Table 1:** Demographic and development assistance characteristics of the BRICS, G7 and G20 member countries

Country	Member of BRICS	Member of G7	Member of G20	Percent of global population (year)	Official Development Assistance -- % of global total (year)	Development Assistance for Health -- % of global total (year)	National level Health expenditure as % of GDP
Argentina			x	0.58% (2017)	NA	NA	NA
Australia			x	0.33% (2018)	2.3% (2016)	1.1% (2016)	9.6% (2016)
Brazil	x		x	2.7% (2018)	0.7% (2010)	NA	NA
Canada		x	x	0.49% (2018)	2.8% (2016)	2.6% (2016)	10.6% (2016)
China	x		x	18.3% (2018)	5.0% (2014)	NA	NA
France		x	x	0.88% (2018)	6.7% (2016)	3.4% (2016)	11.0% (2016)
Germany		x	x	1.1% (2017)	17.3% (2016)	3.9% (2016)	11.3% (2016)
India	x		x	17.5% (2018)	0.94% (2014)	NA	NA
Indonesia			x	3.4% (2018)	NA	NA	NA
Italy		x	x	0.78% (2018)	3.5% (2016)	0.7% (2016)	8.9% (2016)
Japan		x	x	1.7% (2018)	7.3% (2016)	2.3% (2016)	10.9% (2016)
Mexico			x	1.6% (2018)	NA	NA	5.8% (2016)
Republic of Korea			x	0.68% (2018)	1.6% (2016)	1.1% (2016)	7.7% (2016)
Russian Federation	x		x	1.9% (2018)	0.8% (2015)	NA	7.1% (2014)
Saudi Arabia			x	0.44% (2018)	2.9% (2016)	NA	4.7% (2014)
South Africa	x		x	0.75% (2018)	0.02% (2012)	NA	8.8% (2014)
Turkey			x	1.1% (2018)	4.5% (2016)	NA	5.4% (2014)
USA		x	x	4.3% (2018)	24.1% (2016)	34.0% (2016)	17.2% (2016)
UK		x	x	0.86% (2018)	12.77% (2016)	10.9% (2016)	9.7% (2016)
European Union totals			x	6.9% (2015)	49.8% (2016)	NA	NA
BRICS, G7 and G20 totals							
	Global population % (year)		Global GDP % (year)	Official Development Assistance % (year)		Development Assistance for Health % (year)	
BRICS	41% (2016)		22% (2016)	7.5% (2010–2015 average)		NA	
G7	11% (2015)		31% (2017)	73% (2016)		57.8% (2016)	
G20	66% (2017)		80% (2017)	89.5% (2010–2016 average)		NA	

*NA* data not available

Data sources [12, 14, 17, 74–83]:

While the inclusion of health in global summitry is not new, it has gained a prominence that reflects the rising salience of health on international agendas. Despite the BRICS, G7 and G20’s financial clout and contributions to development assistance, scholarly analyses of the major players influencing the global health agenda have, to date, largely focused on multilateral and global health institutions such as the WHO, World Bank, USAID and others [21, 24–27]. While some have investigated the influence of financial power groups including the BRICS and G7 [5, 28, 29], these analyses have explored the clubs’ motivations, agenda-setting in global health governance, individual compliance with their summit commitments, and contributions to shaping the SDGs [9, 11, 19, 30–32]. Little research has compared and contrasted the BRICS, G7 and G20’s health priorities, particularly after the adoption of Agenda 2030 in September 2015.

In this review, we briefly explore the recent history of global health in foreign policy before comparing the health priorities of the BRICS, G7 and G20 to the health targets set in the 2030 Agenda for Sustainable Development. We use the 2030 Agenda as it represents the globally agreed blueprint and commitments for all UN member states, and stated commitments to the Agenda are, we believe, important to track among those country groupings (“clubs”) yielding both soft and hard power in global diplomacy. We analyzed publicly available communiqués from the respective clubs’ meetings of health ministers. We reviewed only those communications published after the adoption of Agenda 2030 in September 2015.

### Recent global health diplomacy

Although there is a long history of cooperation between and among countries on preventing the spread of infectious disease, the period since 2000 has seen a rise in the efforts of global health diplomacy addressing a range of health issues. After the adoption of the Millenium Development Goals (MDGs) [33], AIDS became the first health issue to be discussed by the UN Security Council in 2001, including with a focus on peacekeeping and HIV prevention [34]. In the same year, the pandemic was the focus of a UN General Assembly special session (a first for a health issue), where heads of state adopted the Declaration of Commitments on HIV/AIDS [35]. Following an initiative of foreign affairs ministers from Brazil, France, Indonesia, Norway, Senegal, South Africa, and Thailand, who in 2007 called for foreign policy dialogue to include health [36], the confluence of global health and foreign policy was raised in the UN General Assembly in 2008 where it is now discussed annually [37]. In 2010 the General Assembly officially adopted a consensus to call greater attention to health as a critical policy issue on the international agenda [38]. The following year, the Security Council and General Assembly discussed HIV again, while the General Assembly subsequently included sessions on non-communicable diseases (NCDs), tuberculosis (TB) and universal health coverage (UHC) on its agenda [39].

**Fig. 1:**
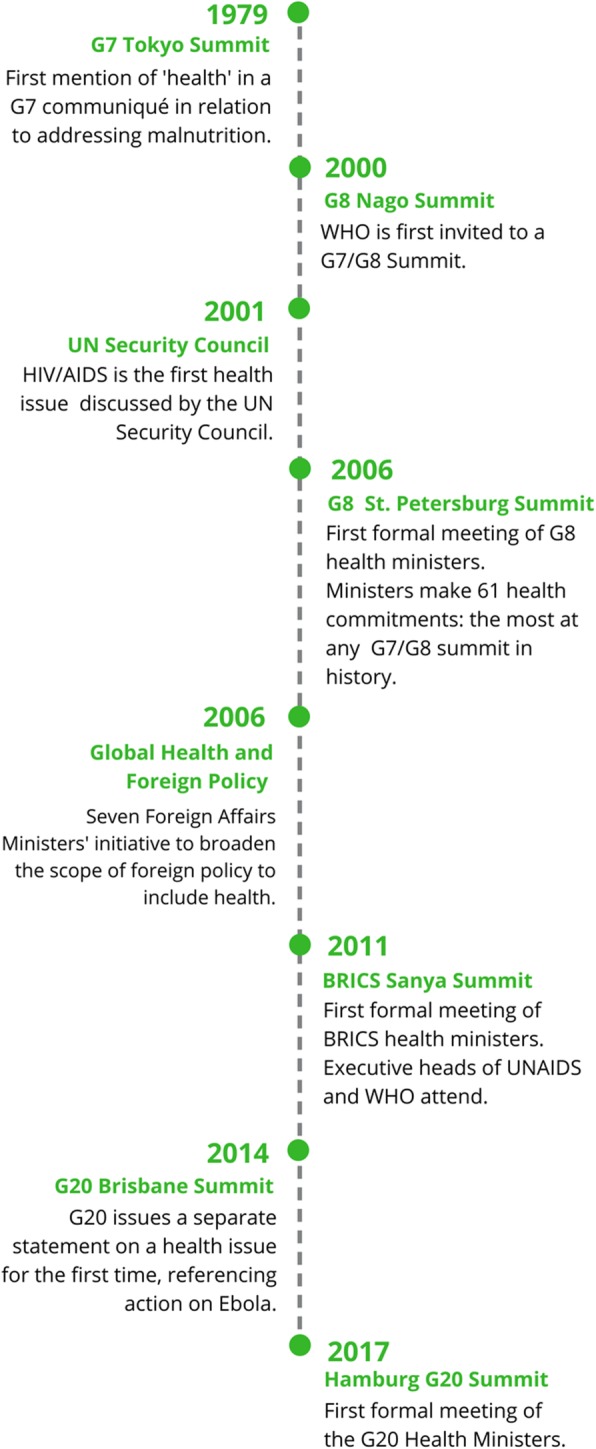
A brief history of global health in recent foreign policy commitments

While the UN provides a forum for global debate, smaller clubs of countries have united around specific health issues which reflect their common interests. Health first appeared in a G7 communiqué from the 1979 Tokyo Summit, referencing cooperation to address malnutrition [40] – see Fig. 1. The G7/G8 made health commitments for two decades before WHO was first invited to the 2000 Nago Summit and before their health ministers first met formally in 2006. G7/G8 health ministers met again in 2008 and annually from 2015 to 2017. BRICS health ministers first met formally in 2011, joined by the Executive heads of WHO and UNAIDS; and have convened annually since 2013. The G20 health ministers met for the first time in May 2017.

Figure 1 data sources [36, 41, 42]:.

The clubs’ heads of state summit declarations reveal their highest priorities. The BRICS leaders’ 2017 Xiamen communiqué stresses enhancing BRICS’ role in global health governance, improving capacity to combat infectious diseases, and enhancing health systems and financing [43]. While in the mid-2000s the G7/G8 made numerous health commitments including financial pledges [6], their 2017 Taormina communiqué features only one paragraph on health security and emergency preparedness and makes no quantitative commitments. The G20’s 2017 Hamburg Leaders’ Declaration devotes one paragraph to health crises and International Health Regulations (IHR) implementation, and another to anti-microbial resistance (AMR) and AMR research and development (R&D).

Independent analyses by the University of Toronto have identified positive compliance among the BRICS, G7 and G20 with regard to their stated commitments, though compliance has varied by topic and member [44, 45]. BRICS countries have demonstrated a positive compliance trend, fulfilling their 2014, 2015 and 2016 summit commitments at rates of 70, 78 and 89% respectively [46]. From 1996 to 2005, the role of the G7/G8 was described as an ‘effective, high-performing’ centre of global health governance [9], and they exponentially increased their health deliberations, decisions, and mobilization of health financing from 2001 to 2005 [9]. Between 1975 and 2009, G7/G8 members complied with commitments at an average level of 77% [47], while the average compliance score of G20 members with their 2008–2011 summit commitments was approximately 70% [30]. These compliance assessments are critical for external accountability and documenting what is achieved pursuant to the summits of political clubs [30] – notably, high compliance has historically translated into health financing and support to development [9, 46].

Given the general reliability of the clubs’ commitments in predicting their actions; their collective economic, political and development assistance power and influence, and the potential for global health leadership and impact through a ‘minilateral’ approach^1^, our study aimed to compare the BRICS, G7 and G20 health priorities against the health targets of Agenda 2030. Our central question was whether these powerful clubs have fully aligned with the SDG health goal (SDG3) and its targets (see Table 2), as this will generally indicate trends in action/resource allocation. Given increasing emphasis on multi-sectoral action and rights-based approaches towards achieving the SDGs [48–50], we also assessed each club’s commitment to the principles and ways of working presented in Agenda 2030.

**Table 2:** Sustainable Development Goal 3 health targets, means-of-implementation targets, and principles and ways of working under Agenda 2030

SDG 3 Health targets	Code definitions
3.1 By 2030, reduce the global maternal mortality ratio to less than 70 per 100,000 live births.	Direct commitment or weak/indirect reference to maternal health or maternal mortality
3.2 By 2030, end preventable deaths of newborns and children under 5 years of age, with all countries aiming to reduce neonatal mortality to at least as low as 12 per 1000 live births and under-5 mortality to at least as low as 25 per 1000 live births.	Direct commitment or weak/indirect reference to child survival (neonatal or child health or mortality)
3.3 By 2030, end the epidemics of AIDS, tuberculosis, malaria and neglected tropical diseases and combat hepatitis, water-borne diseases and other communicable diseases.	Direct commitment or weak/indirect reference to infectious diseases (HIV/AIDS, tuberculosis, or malaria; neglected tropical diseases; or hepatitis, water-borne and other communicable diseases)
3.4 By 2030, reduce by one third premature mortality from non-communicable diseases through prevention and treatment and promote mental health and well-being.	Direct commitment or weak/indirect reference to non-communicable diseases or mental health
3.5 Strengthen the prevention and treatment of substance abuse, including narcotic drug abuse and harmful use of alcohol.	Direct commitment or weak/indirect reference to substance abuse (narcotic drug abuse or alcohol abuse)
3.6 By 2020, halve the number of global deaths and injuries from road traffic accidents.	Direct commitment or weak/indirect reference to road traffic injuries
3.7 By 2030, ensure universal access to sexual and reproductive health-care services, including for family planning, information and education, and the integration of reproductive health into national strategies and programmes.	Direct commitment or weak/indirect reference to access to sexual or reproductive health care services
3.8 Achieve universal health coverage, including financial risk protection, access to quality essential health-care services and access to safe, effective, quality and affordable essential medicines and vaccines for all.	Direct commitment or weak/indirect reference to universal health coverage or access to medicines
3.9 By 2030, substantially reduce the number of deaths and illnesses from hazardous chemicals and air, water and soil pollution and contamination.	Direct commitment or weak/indirect reference to addressing environmental pollution or contamination
SDG 3 Means-of-implementation targets	
3.a Strengthen the implementation of the WHO Framework Convention on Tobacco Control in all countries, as appropriate.	Direct commitment or weak/indirect reference to the WHO FCTC or tobacco restrictions
3.b Support the research and development of vaccines and medicines for the communicable and non-communicable diseases that primarily affect developing countries, provide access to affordable essential medicines and vaccines, in accordance with the Doha Declaration on the TRIPS Agreement and Public Health, which affirms the right of developing countries to use to the full the provisions in the Agreement on Trade-Related Aspects of Intellectual Property Rights regarding flexibilities to protect public health, and, in particular, provide access to medicines for all.	Direct commitment or weak/indirect reference to investment and support of research & development for diseases of the developing world; or to access to medicines via TRIPS
3.c Substantially increase health financing and the recruitment, development, training and retention of the health workforce in developing countries, especially in least developed countries and small island developing States.	Direct commitment or weak/indirect reference to health financing or workforce (increasing health financing or supporting human resources for health in low & middle-income countries)
3.d Strengthen the capacity of all countries, in particular developing countries, for early warning, risk reduction and management of national and global health risks.	Direct commitment or weak/indirect reference to early warning, risk reduction and management of national & global health risks
SDG 3 Principles	
Commitment to the Sustainable Development Goals	Explicit commitment to the SDGs
The right to health; rights-based approaches; and human rights	Direct commitment or weak/indirect reference to the right to health or human rights
Leaving no-one behind	Explicit reference to leaving no-one behind, or direct commitment or weak/indirect reference to sexual or ethnic minorities, vulnerable populations, or refugees, migrants or internally displaced people
Equity/equality	Direct commitment or weak/indirect reference to addressing inequality, gender equality, women’s empowerment, or non-discrimination
SDG 3 Ways of working	
Inter-sectoral collaboration	Direct commitment or weak/indirect reference to inter-sectoral collaboration or multidisciplinary cooperation
Engagement with non-state actors	Direct commitment or weak/indirect reference to working with other sectors and actors
Addressing the social determinants of health (SDOH)	Direct commitment or weak/indirect reference to the social determinants of health

Data sources [84, 85]

## Methods

We used content analysis to review the health ministerial communiqués issued by the political clubs after the SDGs were adopted at the UN General Assembly of September 2015. These include the BRICS (Delhi, December 2016; Tianjin, July 2017), G7 (Berlin, October 2015; Kobe, September 2016; Milan, November 2017), and G20 (Berlin, May 2017) – six communiqués in total. The first author coded each communiqué one paragraph at a time, applying deductive codes according to the nine health targets; four ‘means-of-implementation’ targets; and seven principles and ways of working for SDG 3 as set out in Agenda 2030 (Table 2), which formed our coding framework. The senior author subsequently checked the applied codes, and the first and senior author discussed any disagreement to ensure consistency of coding.

Informed by Kirton et al.’s summit commitment reference manual [51], including its criteria for quality of summit commitments, definition of commitment, and scoring methods [52], we compared the clubs’ commitments against one another and against the Agenda 2030 nine health targets; four ‘means-of-implementation’ targets; and seven principles and ways of working for SDG 3. We issued a green light if a club made a direct reference/commitment to the target in at least one communiqué; an amber light for weak/indirect references; and a red light if a club omitted the target entirely from all communiqués.

## Results

### Commitments of BRICS, G7 & G20 to SDG 3 health and means of implementation targets

**Fig. 2:**
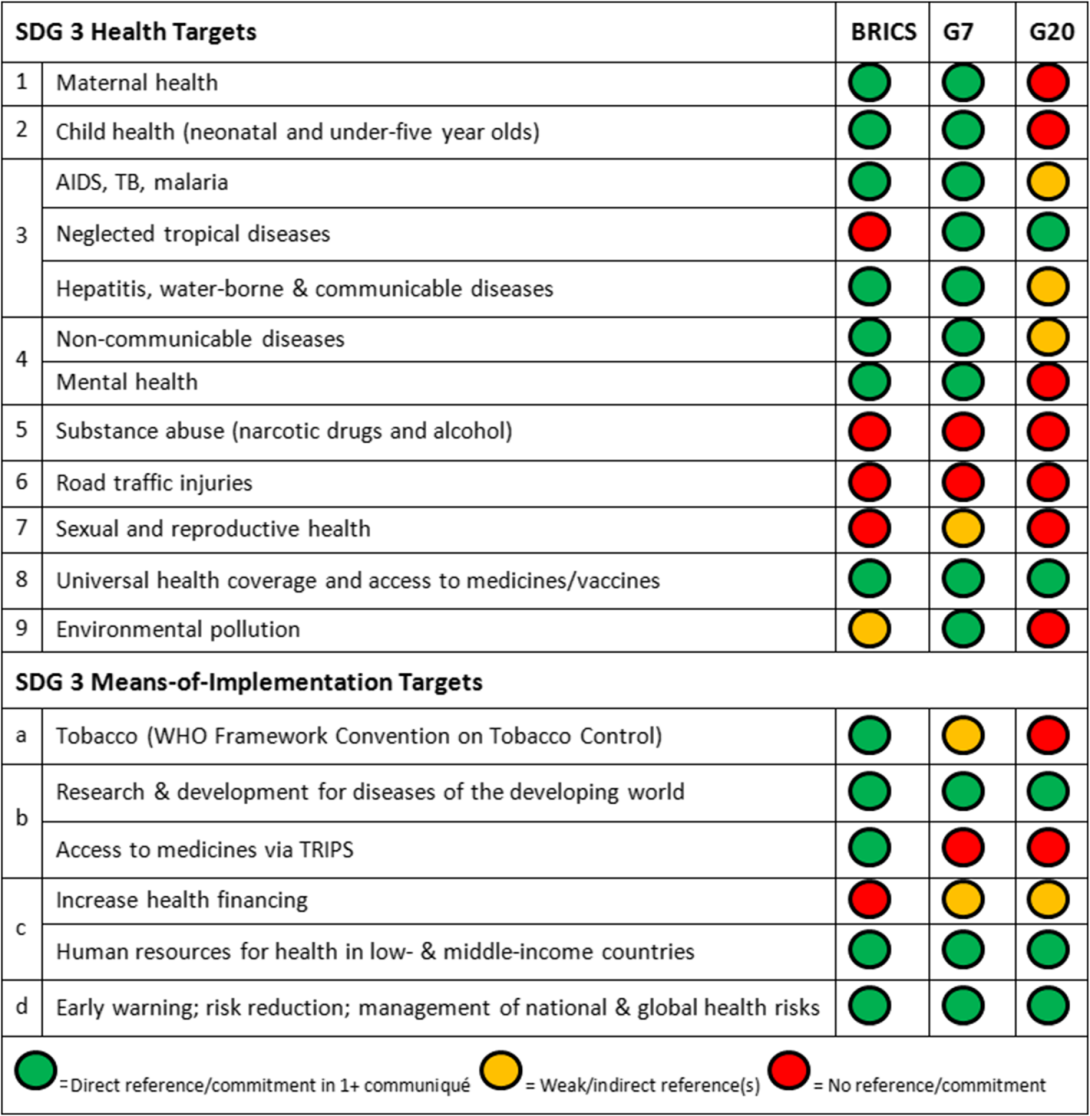
The BRICS, G7 and G20 Health Ministers’ commitments to the targets of SDG 3. BRICS: 6th Health Ministers’ Meeting Communiqué, Delhi, India, December 2016; 7th Health Ministers’ Meeting Communiqué, Tianjin, China, July 2017. G7: Declaration of the G7 Health Ministers, Berlin, Germany, Oct 2015; Health Ministers’ Meeting Communiqué, Kobe, Japan, September 2016; Health Ministers’ Meeting Communiqué, Milan, Italy, November 2017. G20: Health Ministers’ Berlin Declaration, Berlin, Germany, May 2017

The BRICS health ministers’ communiqués average 1559 words, the G7 communiqués average 3408 words, and the single G20 communiqué is 3977 words in length; with each club describing their commitments with similar specificity. In general, the communiqués do not speak to individual countries’ priorities, but rather document the issues and actions prioritized by the countries within the clubs as a unified political bloc [53–57] Fig. 2.

#### SDG 3.1: maternal mortality

BRICS “emphasized the importance of child survival through progressive reduction in maternal mortality” [53], while the G7 “will pay particular attention to maternal, newborn, and child health” and emphasized “context-specific investments in evidence-based interventions that address the root causes of mortality, morbidity, discrimination and violence” [54]. The G20 did not address maternal mortality.

#### SDG 3.2: child survival

BRICS highlighted child survival through “progressive reduction in neo-natal mortality, infant mortality, and under-5 mortality with the aim of achieving the unfinished agenda of the Millennium Development Goals and the relevant SDGs” [56]. The G7 “recognize the importance of addressing childhood malnutrition” [55], but did not mention child survival. The G20 made no mention of child health or survival.

#### SDG 3.3: infectious diseases

BRICS made strong commitments on AIDS, TB and malaria and referenced the 90–90-90 HIV treatment targets [56], but did not mention neglected tropical diseases (NTDs) or hepatitis. The G7 asserted “we have committed to end the epidemics of AIDS, TB, malaria and NTDs by 2030” [55], highlighted polio eradication [54], and emphasized water, sanitation and hygiene measures to combat infectious diseases. The G20 highlighted drug-resistant TB as a threat; mentioned addressing NTDs through UHC and infectious diseases through improving sanitation and immunization [58], but did not make commitments on AIDS, malaria, NTDs or hepatitis.

#### SDG 3.4: non-communicable diseases (NCDs) and mental health

BRICS mentioned strengthening services to combat NCDs, highlighted the importance of addressing their risk factors [53], and agreed to make collaborative efforts “to achieve the target of reduction in premature mortality due to NCDs by one-third by 2030 as per SDG Target 3.4” [56]. The G7 mentioned preventing deaths and disabilities caused by NCDs through addressing environmental pollution [54], and raised concerns about NCDs throughout the life course and “their impact on the quality of life of the elderly” [55]. The G20 acknowledged that strong and resilient health systems are the basis for the effective prevention and control of non-communicable and communicable diseases [58].

With regard to mental health, BRICS “agreed to cooperate for combating mental disorders through a multi-pronged approach” including a mental health policy, a life cycle approach, sharing of innovations in mental health promotion, and exchange of best practices [56], while the G7 emphasized improving access to mental health services amongst migrants, refugees, women and adolescents [54]. The G20 did not reference mental health.

#### SDG 3.5 and SDG 3.6: substance abuse and road traffic injuries

Neither of these issues was mentioned in any of the communiqués reviewed.

#### SDG 3.7: sexual and reproductive health care services

The G7 affirmed that “sustainable and equitable health systems will better respond to lay a foundation for achieving UHC, which better prepares health systems to respond to diverse health challenges, including reproductive health” [55]. Neither BRICS nor G20 mentioned sexual or reproductive health, and no club mentioned sexual health.

#### SDG 3.8: universal health coverage

BRICS recognized that “promoting access to medicines and vaccines, in particular essential medicines, that are affordable, safe, efficacious, and of quality, is imperative for the right of everyone to the enjoyment of the highest attainable standard of physical and mental health” [56] and welcomed the recommendations of the UN High Level Commission on Health Employment and Economic Growth towards delivering UHC. The G7 explicitly cited SDG 3.8; reiterated “the importance of strengthening health systems through each country’s path towards Universal Health Coverage”; and emphasized inclusive health services [54]. The G20 highlighted functioning health systems and access to affordable basic care as essential to global health, acknowledged that “strong and resilient health systems will contribute to UHC” and welcomed partnerships towards these goals [58].

#### SDG 3.9: environmental pollution and contamination

BRICS mentioned “environmental health” in relation to cooperation on surveillance workshops [56], but did not address pollution. The G7 “acknowledge that some environmental-related factors contribute to health risks”; asserted “it is crucial to decrease exposure to air pollution, including by reducing emissions in urban areas”; and that it “will support inter-sectoral, evidence-based foresight exercises and policies to reduce drivers of pollution concentrations, and promote innovative solutions” [54]. The G20 did not mention pollution concerns.

#### SDG 3 (a): WHO framework convention on tobacco control (FCTC)

BRICS explicitly referenced the FCTC, and “renewed their commitment to the Convention, both as a public health treaty and as a Goal under Agenda 2030 for Sustainable Development.” [56] The G7 mentioned improving indoor air quality through restrictions on tobacco smoking [54], but neither the G7 nor G20 referenced the FCTC.

#### SDG 3 (b): research and development (R&D) for diseases of the developing world

BRICS “agreed to jointly promote R&D of drugs, vaccines, diagnostics and medical technologies, including through the creation of a R&D consortium on TB, HIV and malaria” [53]. BRICS emphasized upholding the guiding principle of de-linkage between R&D costs and the price of health products, and “the full use of TRIPS flexibilities” as well as “protecting policy space against TRIPS plus provisions and other measures that impede access to medicines” [56]. The G7 “recognized the role of R&D in improving health and health systems” [55], and called to promote resource mobilization towards R&D. The G20 recognized R&D as necessary for “the timely availability and development of quality medicines, vaccines, and diagnostics”; called for coordination in research efforts; emphasized sustainable funding; and raised concerns that R&D for AMR is insufficient [58]. Neither the G7 nor G20 alluded to TRIPS provisions for access to medicines.

#### SDG 3 (c): health financing and workforce

BRICS did not address health financing but committed to inter-BRICS cooperation “for capacity development of human resources in public health and clinical medicine.” [56] The G7 “acknowledge that WHO’s financial and human resource capacities have to be strengthened” and called to address health workforce shortages, poor health financing by countries [54] and developing human resources for health emergencies, but made no concrete commitments. The G20 “recognize the importance of sustainable financing for health systems” and support to developing countries in health system strengthening. The G20 also encouraged investments towards skilled health workforces, but emphasized that member states “should strive to meet health personnel needs with their own human resources” [58].

#### SDG 3 (d): early warning, risk reduction and global health risk management

BRICS recognized the importance of monitoring disease outbreaks and enhancing “the cooperation of institutions under the mechanism of Global Outbreak Alert and Response Network” [53] in light of the IHR. The G7 welcomed efforts to “establish standard operating procedures for health and humanitarian system-wide coordination to respond to global public health emergencies” [55] and called for effective IHR implementation. The G20 referenced the primary focus of the 2017 German G20 Summit Presidency on mitigating health emergencies. It thus recognized linkages between disease outbreaks, AMR and the global economy; called for a coordinated response, joint international commitments, and IHR compliance; and emphasized well-trained personnel to respond to crises [58]. All three clubs emphasized AMR in the context of global health risk management: BRICS “recognized that AMR including in diseases such as TB and HIV/AIDS, seriously threatens public health, and economic growth, reiterated to support the suggestions of United Nations high-level meeting on antimicrobial resistance” [53], while the G7 the G20 each allocated several paragraphs to the importance of addressing AMR [54, 57].

#### Omitted SDG 3 health priorities

SDG targets 3.5 (substance use including narcotics and alcohol), 3.6 (road traffic injuries and deaths), and 3.7 (sexual and reproductive health) were absent from all three communiqués, with the exception of the G7 mention of reproductive health. These omissions are significant given the high attributable burden of these conditions to Disability Adjusted Life Years (DALYs), Years of Life Lost (YLL) and mortality globally. For example, road traffic accidents accounted for 3.0% of total global Disability-Adjusted Life Years (DALYs) - 4.7 times the total global DALYs for breast cancer - in 2016. These figures vary widely by country - for example, over 4% of DALYs are attributable to road accidents in Brazil, China and South Africa [59].

### Commitments of BRICS, G7 & G20 to SDG3 principles and ways of working (Fig. 3)

#### Explicit commitment to the SDGs

BRICS “comitted to strengthen intra-BRICS cooperation…to achieve the 2030 Sustainable Development Agenda” [53]. The G7 asserted “we are fully committed to implementing the health-related SDGs” [55]; while the G20 “reaffirm our commitment to achieve the health-related goals and targets of the 2030 Agenda for Sustainable Development” [58].

**Fig. 3:**
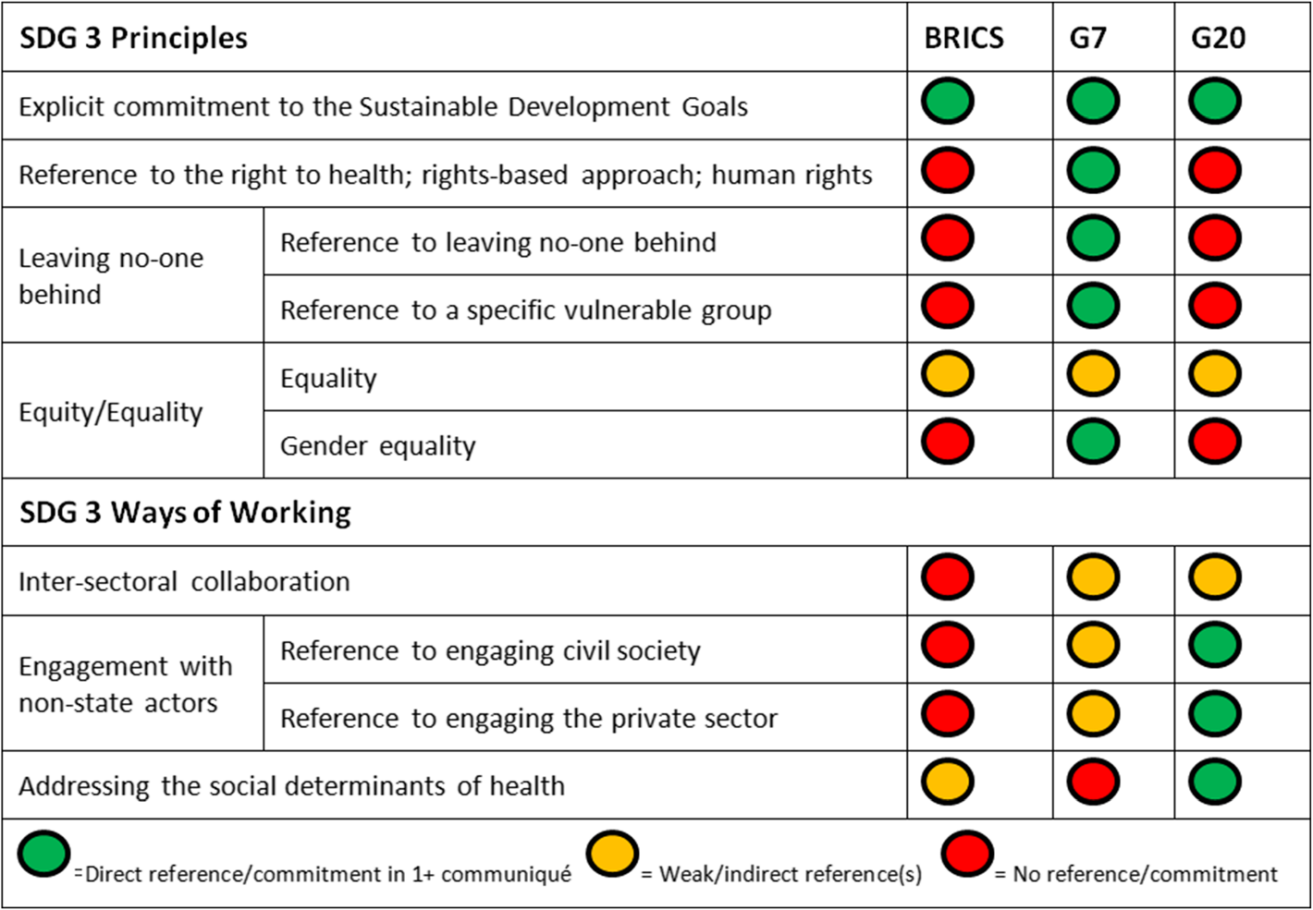
The BRICS, G7 and G20 Health Ministers’ commitments to the principles of SDG 3. BRICS: 6th Health Ministers’ Meeting Communiqué, Delhi, India, December 2016; 7th Health Ministers’ Meeting Communiqué, Tianjin, China, July 2017. G7: Declaration of the G7 Health Ministers, Berlin, Germany, Oct 2015; Health Ministers’ Meeting Communiqué, Kobe, Japan, September 2016; Health Ministers’ Meeting Communiqué, Milan, Italy, November 2017. G20: Health Ministers’ Berlin Declaration, Berlin, Germany, May 2017

#### The right to health; rights-based approaches; and human rights

BRICS referenced international human rights law and policy incoherence on access to medicines, but did not otherwise cite the right to health. The G7 affirmed “the enjoyment of the highest attainable standard of health is one of the fundamental rights of every human being” [60], and highlighted “women’s and adolescents’ rights related to health and health care” [54]. The G20 mentioned “respecting privacy and all other human rights with regard to all collected health data” [58], but did not mention rights in other contexts.

#### Leaving no-one behind

The G7 affirmed that “support for migrants and refugees should consider their specific needs, leaving no one behind” [54]; and mentioned adolescents, women, immigrants, refugees and the elderly as vulnerable groups, emphasizing that health systems should be responsive to their specific needs [55]. The G20 emphasized providing health care “to all and without discrimination” [58] but neither the G20 nor BRICS referenced leaving no-one behind or a specific vulnerable group.

#### Equity/equality

BRICS referenced equity issues in relation to access to anti-microbials [53]. The G7 stated “we seek to reduce global inequalities” [54], while the G20 highlighted that “support to developing countries in strengthening their health systems… would increase their capacity to reduce health inequities” [58].

The G7 made several direct appeals to women’s rights and empowerment, asserting “we commit to respecting, protecting and fulfilling women’s right to the enjoyment of the highest attainable standard of physical and mental health, without discrimination. We will take concrete actions to strengthen health systems, policies, laws and programs that support their empowerment.” [54] The G7 also emphasized promoting women’s equal opportunities; participation in decision-making processes, and economic participation. The G7 alone condemned sexual and gender-based violence against women and girls, and recognized other genders by calling to actively involve men and boys as agents of change [54]. Neither BRICS nor G20 referenced gender equality.

#### Inter-sectoral collaboration

The G7 called to promote inter-sectoral coordination towards achieving vector control in the context of health risks [54] and AMR [60]. BRICS and the G20 did not reference collaboration between sectors.

#### Engagement with non-state actors

BRICS did not mention civil society or the private sector. The G7 mentioned engaging with “community sectors” and cooperating with the private sector in relation to health security [54] and addressing AMR [60]. The G20 acknowledged “the role of the public and private sectors and civil society” in strengthening health systems worldwide [58].

#### Addressing the social determinants of health (SDOH)

BRICS referenced continued research into SDOH as they relate to the implementation of the FCTC [56]. The G20 reiterated “our determination to take action on SDOH as reflected in resolution WHA [World Health Assembly] 62.14 ‘*Reducing health inequities through action on the social determinants of health’*” [58]. The G7 did not reference SDOH.

## Discussion

The global health leadership of the BRICS, G7 and G20 represents an exercise of soft power, defined as applying economic and cultural clout to shape the preferences of other actors [11, 61]. The health issues prioritized by the major economies of the BRICS, G7 and G20 members are likely to have influence not only within their own domestic sphere, but also across other countries and actors. Given that the G7 collectively contributes approximately 58% to global development assistance for health and the G20 contributes approximately 90% to official development assistance, the positions taken by these political clubs potentially impact on the direction of development assistance agendas and programmes in the health sector globally.

The G7 featured the most comprehensive coverage of the SDG 3 targets and principles, making direct commitments on 17 of 28 areas, and also had the greatest proportion of amber lights [7] representing weak or indirect references. The G20 had the least comprehensive coverage, with 13 of 28 areas omitted entirely. With regard to the 13 SDG 3 health and means-of-implementation targets specifically, the BRICS featured the same coverage of targets as the G7 (12 green lights each), despite BRICS’ communiqués featuring half as many words as the G7’s on average.

All three clubs placed considerable weight on health emergencies and AMR often framing them in terms of their potential impact on global trade and the economy, but made fewer commitments on NCDs and environmental pollution despite their major impact on health and wellbeing around the world, as well as their increasing influence on economic growth and productivity [62–64]. Each club elevated the salience of UHC in strengthening systems to prepare for and respond to disease outbreaks.

The analysis reveals key differences across the clubs in terms of priorities. BRICS emphasized their commitment to the WHO Framework Convention on Tobacco Control and the full use of TRIPs flexibilities to protect access to medicines, in contrast to the G7 and G20 which did not reference either tobacco control nor TRIPs nor the agreement’s impact on public health. The G7 explicitly highlighted environmental health, human rights and gender equality issues, all of which were omitted by BRICS and the G20. The G20 alone made references to engaging civil society and private sector actors and addressing SDOH.

The clubs’ collective lack of attention to substance use and road traffic morbidity and mortality is concerning due to their significant burden of ill-health and economic impact. In 2016, substance use (alcohol and narcotics) disorders accounted for 3.1% of all years lived with a disability (4.3% among males; 2% among females) [59] . Current substance use treatment costs are $35bn annually despite only 16% of problem substance users receiving necessary treatment [65], and substance use remains overlooked in development dialogues despite its influence on countries’ progress across Agenda 2030 [65, 66]. In 2016, road traffic injuries accounted for 2.45% of total deaths (3.4% among males; 1.3% among females), and were the leading cause of DALYs due to injury [59]. In low- and middle-income countries (LMICs), road traffic injuries and deaths were estimated to cause economic losses of up to 5% of GDP in 2015 [67]. As these health and economic burdens are considerable, the exclusion of substance use and road traffic injuries represent a large gap in the health-related commitments of the BRICS, G7 and G20.

While the BRICS and G7 have recognized the importance of NCDs, it seems none of the clubs prioritise the epidemic in a proportionate way to its true costs. Over the period 2011–2025, the global cumulative lost output associated with the most common NCDs (diabetes, cardiovascular and respiratory diseases, cancer) is estimated at over US$ 47 trillion [68]. Given that NCDs are the leading cause of death and disability globally and that two thirds of NCD deaths are associated with tobacco and alcohol use, poor diet and physical inactivity [69], addressing NCDs and their related social and commercial determinants of health should be a priority for all actors exerting influence over the global health agenda.

Despite the BRICS, G7 and G20’s commitments to much of the SDG 3 agenda in their respective communiqués, the clubs’ assertions are weak in so far as formulating tangible obligations or actions. Kirton et al. propose discreteness, specificity (i.e., measurability), future orientation, ambition, and timeliness as important factors which inform the quality of commitments [51]. Within this framework, the BRICS, G7 and G20 score highly if unevenly on ambition in that many commitments indicate an aspiration that would move global health from the status quo, but more often than not use the language of ‘recognize’, ‘emphasize’ and ‘acknowledge’. The concerns are largely timely in addressing issues of considerable urgency (i.e., global health emergencies and AMR). All three clubs, however, score low on specificity as many of their commitments are described in language which does not clearly identify the target of the action (and in some cases encouraging others to act – e.g., on raising domestic resources) nor do the communiqués include concrete measures for reporting or accountability.

The general omission of measurable quantitative targets across the BRICS, G7 and G20 communiqués is especially concerning given historical precedents, particularly with regard to the G7/G8 which made explicit financial commitments during the mid-2000s – commitments which were highly influential in the global health landscape. For example, the G8’s ability to leverage increased funds for specific health targets was illustrated through the launch of the Global Fund to Fight AIDS, Tuberculosis and Malaria over the 2000 and 2001 summits, which raised over US $31bn, and its 2007 summit drew US $60bn in commitments for infectious diseases [28]. In 2010, the G8 heads of state committed US $5bn to the Muskoka Initiative on Maternal, Newborn and Child Health [70]. While all three political clubs have publicly recognized the importance of Sustainable Development Goal 3, none has made quantitative commitments, financial or otherwise, for health in their communiqués since the adoption of the Agenda 2030 implementation.

Finally, given the clubs’ primary aim of global economic governance and enhancing economic growth within a neoliberal political framework, it is perhaps unsurprising that equity concerns were not overtly mentioned by any political club. Of concern, the general lack of commitment to action on SDOH within all clubs’ communiqués (with exception of a brief reference from the G20) raises important questions regarding the strength of these countries’ commitments to the shared global health vision of Agenda 2030. The Agenda 2030 targets are interdependent and indivisible, and will not be achieved without action on SDOH [71] - thus the broad omission of the SDOH within the communiqués raises concerns around the potential for effective and sustainable improvements in population health.

Our paper builds upon prior analyses of the BRICS, G7 and G20’s roles and performance in global health governance, including assessment of the quality of these clubs’ and their respective countries’ motivations for engagement in global health negotiations, their leveraging of soft power, actions in agenda-setting, and individual compliance with health commitments [9, 11, 19, 30]. Where analyses have compared the clubs, this has generally been in relation to health spending patterns within countries [16], performance of their summits [31], and compliance to summit commitments [30]. We aimed to extend these analyses by focusing explicitly on health communiqués, and assessing these within the context of the SDGs. Previous work has shown that commitments from these political clubs generally translate into resource allocation within development assistance for health, thus highlighting the importance of identifying priority and neglected SDG3 target areas within the clubs’ communiqués. Our paper has shown clear areas of divergence between the SDG3 targets and the priorities identified by these political clubs, and has highlighted those health areas and underlying principles that have seemingly been overlooked by the clubs to date.

Given the gaps identified in the BRICS, G7 and G20 commitments with regard to SDG 3, we propose a five-point agenda that health ministers, their advisors and health advocates consider in elaborating future communiqués (Table 3).

**Table 3:** Recommendations towards aligning the global health leadership of the BRICS, G7 and G20 with Agenda 2030 for Sustainable Development

1. Attend to the neglected SDG 3 health targets *Explicit commitments in the areas of substance use, road traffic accidents and sexual health are needed to address their associated burdens of ill-health and to promote leadership in these areas.*	
2. Place greater emphasis on upstream determinants of health *Greater attention to the social, commercial and environmental determinants of health as well as the prevention of disease will enhance progress towards shared goals of sustainable economic development.*	
3. Ensure commitment to equity and leaving no-one behind *Consider the vulnerable groups and populations who are least likely to benefit from current forms of economic and development activities, and push for novel approaches which leave no-one behind.*	
4. Adopt explicit commitments to rights *Place human rights, including the right to health, at the centre of commitments as well as place greater emphasis on the right to participation.*	
5. Make higher quality commitments which include accountability mechanisms. *Make time-bound, quantitative and financial commitments to health and development goals in line with the SDGs, and create accountability mechanisms between member countries within each political club to promote shared responsibility.*	

## Limitations

This study features several limitations. We analyzed only six health ministerial communiqués in total, which for the G7 and BRICS do not represent the entirety of their public health communiqués. These 6 health communiqués are unlikely to represent the complete health aims, objectives and strategy for any of the single political institutions, but the small numbers represent only those communiqués issued since Agenda 2030 was signed in September 2015.

The health ministerial communiqués present each political club’s health priorities as a unified political bloc, but do not disaggregate their priorities by individual countries within the club, nor individual recipient countries outside the club. Thus, while these communiqués represent a strong proxy for the shared global health aims of the BRICS, G7 and G20 respectively, they do not permit an analysis of whether the policies and health targets outlined in the documents reflect the individual countries’ priorities, and it is possible that the SDG 3 targets which the clubs perceive best prioritized on a country level may be excluded from their collective health governance aims. Finally, we have criticized the health communiqués for failing to commit to the social determinants agenda. It could be argued that such commitments are made in other sectoral communiqués. While that might be the case, it is arguably the case that the health sector would lead on coordination of SDOH agendas.

## Conclusions

There has been a great deal of attention paid to the role of multilateral, bilateral and private actors in the realm of global health governance, but less attention has so far been focused on important political clubs as represented in the membership of the BRICS, G7, and G20. Our analysis of communiqués has found that health issues are included in the agendas of these political blocs. This is commendable and should provide some hope for the global health community since the commitments, areas for cooperation and priorities signaled by their health communiqués are likely to have wider global health significance.

The political clubs are largely aligned with the targets of SDG3 and this is likely to provide further impetus to achieving Agenda 2030. However, it is of concern that significant burdens of ill health and their social, economic and commercial determinants are neglected by these economic leaders. A broadening of the clubs’ agendas to reflect the evidence on the global burden of ill-health, with greater attention to issues of structural determinants, principles of equity and approaches based in the recognition and realization of human rights, will promote the alignment of these clubs’ aims with the Agenda 2030 vision of health and well-being for all—which leaves no one behind.

## Data Availability

The communiqués upon which this analysis and its conclusions are based are all publicly available from the BRICS, G7 and G20.

## Abbreviations

AIDS: Acquired immunodeficiency syndrome
AMR: Anti-microbial resistance
DALY: Disability-adjusted life year
FCTC: Framework Convention on Tobacco Control
IHR: International Health Regulations
NCD: Non-communicable disease
NTD: Neglected tropical diseases
R&D: Research and development
SDGs: Sustainable Development Goals
SDOH: Social determinants of health
UHC: Universal Health Coverage
UNAIDS: United Nations Joint Programme on HIV/AIDS
WHO: World Health Organization

## Footnotes

1 Minilateralism is described as “the smallest possible number of countries needed to have the largest possible impact on solving a particular problem” [72], even if this raises questions about legitimacy (i.e., whether these smaller groups of countries constitute legitimate fora [relative to the UN] for determining health policies and priorities) [73].
